# Evaluation of proton pump inhibitor administration in hospitalized dogs in a tertiary referral hospital

**DOI:** 10.1111/jvim.16491

**Published:** 2022-07-22

**Authors:** Samantha Duxbury, Emily Sorah, M. Katherine Tolbert

**Affiliations:** ^1^ Department of Clinical Sciences North Carolina State University, College of Veterinary Medicine Raleigh North Carolina USA; ^2^ Department of Small Animal Clinical Sciences, College of Veterinary Medicine and Biomedical Sciences Texas A&M University College Station Texas USA; ^3^ Present address: School of Veterinary Medicine, University of Pennsylvania Philadelphia Pennsylvania USA

**Keywords:** acid suppressant, canine, gastroprotectant, omeprazole, pantoprazole

## Abstract

**Background:**

Although proton pump inhibitors (PPIs) are commonly administered to hospitalized dogs, prescribing patterns and appropriateness of use require continued investigation.

**Hypothesis/Objective:**

Describe prescription patterns and appropriateness of use associated with PPIs in hospitalized dogs at a single tertiary care facility. We hypothesized that the majority of prescriptions would not comply with current guidelines for the rational use of acid suppressants.

**Animals:**

Two hundred randomly selected hospitalized dogs.

**Methods:**

Retrospective evaluation of the medical records associated with a randomly selected sample of hospitalized dogs that received PPIs between January 2013 and December 2018.

**Results:**

A total of 12 610 dogs were admitted for first‐time hospitalization between January 2013 and December 2018. Forty percent of these dogs (5062/12610) were prescribed a PPI PO or IV. Of the 200 randomly selected records, an adequate indication for use was identified in 27% of dogs (54/200). Of the dogs surviving to discharge, 54% (95/175) were discharged with a PPI and 51.6% (49/95) of those were prescribed an inadequate dose.

**Conclusions and Importance:**

Our findings support other studies in which the majority of PPI prescriptions for hospitalized dogs at a tertiary care hospital lacked an appropriate indication. Furthermore, analysis of the prescribing patterns of dispensed PPIs identified a frequent occurrence of dosages considered inadequate, raising concern for ineffective treatment even with appropriate indications of use. With growing concern of adverse effects associated with PPI and other acid suppressant administration in human and veterinary medicine, rational use of these medications following consensus guidelines should be emphasized and treatment should be reserved for dogs with historical, physical examination, clinicopathologic, and imaging findings supportive of an appropriate indication for use.

**Abbreviations**
GI: gastrointestinal
GUE: gastroduodenal ulceration and erosion
H2RA: H2‐receptor antagonist
NSAID: nonsteroidal anti‐inflammatory drug
PPI: proton pump inhibitor

## 1 INTRODUCTION

The World Health Organization defines rational use of medication using 4 criteria: (a) appropriate medication selection based on patient diagnosis; (b) prescription of the medication at a dose expected to be adequate for treatment; (c) prescription of the medication for an amount of time expected to be adequate for treatment; and (d) prescription of the medication at the lowest cost reasonably achievable.^1^

Proton pump inhibitors (PPIs) are effective when used appropriately for the treatment of acid‐related disorders in both human and veterinary medicine. Despite prescribing guidelines in human medicine, in multiple studies, inappropriate prescription patterns characterized by the overuse of PPIs have been identified.^2^, ^3^ Indeed, PPIs are one of the most commonly prescribed drugs in the United States in human medicine and treatment comes at a considerable cost, with an estimated 78.9 billion dollars spent on this class of drugs between 2007 and 2011.^4^

Use of PPIs without clear indications despite increased risk for adverse effects also is considered common in veterinary medicine as determined by other studies.^5^, ^6^, ^7^, ^8^ Our objective was to describe the prescription patterns and appropriateness of PPI administration in hospitalized dogs at a single tertiary care facility. We hypothesized that the majority of prescriptions would not comply with current guidelines regarding appropriate indication of PPI use.^9^

## 2 MATERIALS AND METHODS

### 2.1 Study design and overview

A retrospective review was conducted of the medical records of dogs hospitalized for a first visit in the intensive care unit (ICU) or intermediate care (IMC) ward over a 5‐year period (January 2013‐December 2018) at North Carolina State University Veterinary Teaching Hospital (NCSU VTH). Electronic medical records were searched for all dogs prescribed pantoprazole, esomeprazole, or omeprazole that were concurrently billed for hospitalization in the ICU or IMC ward. A total of 5062 dogs were identified. Of these cases, the medical records of 200 hospitalized dogs were randomly selected. The eligible cases were sorted chronologically based on date of admission. The first and tenth dogs were selected and then every 50th record was reviewed. The following data were collected: (a) signalment, (b) admitting service, (c) PPI administered, (d) primary diagnosis identified, (e) concurrent disease, (f) survival to discharge, (g) outpatient prescription of PPIs, (h) concurrently prescribed medications, (i) dose of outpatient prescription, (j) instructions for outpatient use, and (k) directions for discontinuation.

### 2.2 Study definitions

Study definitions were primarily determined using the American College of Veterinary Internal Medicine (ACVIM) consensus statement.^9^ Appropriate indication for use was defined as history (eg, signs of dysphagia, position of relief), physical examination findings (eg, painful abdomen with compatible history of possible gastric ulceration), imaging (eg, refluxate in esophagus) or clinicopathologic data supportive of the following disease processes: (a) reflux or erosive esophagitis, (b) upper gastrointestinal (GI) bleeding or acute abdomen secondary to gastroduodenal ulceration and erosion (GUE), (c) gastric or duodenal perforation, or (d) prophylaxis for mast cell disease or gastrinoma, and (e) known or suspected nonsteroidal anti‐inflammatory drug (NSAID) toxicosis and not as a preventative measure during routine NSAID use. Unknown indication for use was defined as known or suspected (a) thrombocytopenia‐induced upper GI bleeding, (b) an intrahepatic portosystemic shunt, and (c) International Renal Interest Society stage IV chronic kidney disease. Questionable indication for use was defined as lack of evidence supporting either an appropriate or unknown indication for use. Cases were collected and initial case definitions were assigned by a rotating small animal intern (SD). Final case definitions were assigned after review with the medical intern and a small animal internist (MKT). Medical records were excluded if they were found to be incomplete. All prescribed medications were recorded according to class. Medication classes included: (a) PPIs, (b) anti‐nausea and anti‐emetic medications, (c) pro‐kinetics, (d) gastro‐protectants (other than PPIs as defined by the ACVIM consensus statement to include antacids, H2‐receptor blockers, misoprostol, and sucralfate^9^), (e) antibiotics, (f) appetite stimulants, (g) immunosuppressive medications (other than corticosteroids), (h) corticosteroids, (i) NSAIDs, (j) analgesics, (k) anti‐platelet drugs, (l) anti‐coagulants, (m) and other medications. Prescriptions of PO PPIs in this population of hospitalized patients also were evaluated. An adequate dose of omeprazole was defined as a dosage ≥0.5 mg/kg PO q12h. Provision of adequate directions for administration were defined as timing of medication in relation to food, length of recommended treatment, and instructions for tapering of medication with prolonged use. Prescriptions that lacked any of these directions were considered inadequate.

## 3 RESULTS

### 3.1 Patient demographics

During the study period, 12 610 dogs were admitted to the ICU or IMC of NCSU VTH for first time hospitalization. Oral or IV PPIs were administered to 40% (5062/12610) of these dogs. Sixty‐one breeds or mixed breed dogs were represented among the 200 randomly selected dogs. The most common breeds represented in the study population were Labrador retrievers (24/200, 12%) followed by mixed breed dogs (12/200, 6%), Yorkshire terriers (11/200 5.5%), dachshunds (10/200, 5%), golden retrievers (9/200, 4.5%), and poodles (9/200, 4.5%). The most common breeds admitted to the hospital for any reason during this time frame were the Labrador Retriever followed by mixed breed dogs, golden retrievers, German Shepherd dogs, Yorkshire terriers, boxers, chihuahuas, and dachshunds. The median age (range) of the 200 study dogs was 8 years (4 months‐17 years). One dog was excluded because of an unknown birth date. Demographic data is presented in Table 1.

**TABLE 1:** Demographic information and case characteristics for 200 dogs prescribed a PPI during first time hospitalization at a tertiary referral hospital

	Number (n)	Percentage (%)
Age (years)		
0‐5	73	36.5
6‐10	65	32.5
>10	61	30.5
Unknown	1	0.5
Breed		
Labrador Retriever	24	12.0
Mixed breed dog	12	6.0
Yorkshire Terrier	11	5.5
Dachshunds	10	5.0
Golden Retrievers	9	4.5
Poodles	9	4.5
Beagle	7	3.5
English Bulldog	6	3.0
American Cocker Spaniel	6	3.0
Pomeranian	6	3.0
Other	100	50
Sex		
Female intact	8	4.0
Female spayed	111	55.5
Male intact	15	7.5
Male castrated	66	33.0
Admitting service		
Cardiology	2	1.0
ECC	24	12.0
Internal medicine	101	50.5
Oncology	8	4.0
Neurology	21	10.5
Soft tissue surgery	44	22.0

*Note*: All variables are presented as number (n) followed by percentage.

### 3.2 Indication for use and inpatient prescription patterns

In the majority of hospitalized dogs, an inappropriate indication for use was identified (137/200, 68.5%). Fourteen percent (19/137) of dogs had clinical signs of vomiting or diarrhea or both, 12% (17/137) dogs had a confirmed diagnosis or mass lesion suggestive of pancreatic or non‐gastric related neoplasia, 10% (14/137) of dogs had suspected pancreatitis, 9% (12/137) of dogs had acute kidney injury, 6% (8/137) of dogs had immune‐mediated hemolytic anemia, 6% (8/137) of dogs had a hepatopathy, 4% (6/137) of dogs had intervertebral disc disease, 4% (6/137) of dogs had an extrahepatic portosystemic shunt, and 4% (5/137) of dogs were admitted because of trauma. An appropriate indication for use was identified in a smaller number of hospitalized dogs (54/200, 27%) and the specific indications are summarized in Table 2. A questionable indication for use represented the smallest proportion of dogs (6/200, 3%). One and one‐half percent (3/200) of dogs were excluded because of incomplete medical records. Of all 200 dogs, 54% (108/200) received pantoprazole only, 41% (82/200) received both pantoprazole and omeprazole, 5% (10/200) received omeprazole only and no dogs received esomeprazole.

**TABLE 2:** Indication for use categories and distribution among 54 dogs prescribed a PPI with an identified adequate indication for use

Adequate indication	Prescriptions (n [%]) n = 54
Reflux and erosive esophagitis	13 (24.1)
Upper GI bleeding or acute abdomen secondary to GUE	34 (63.0)
Gastric or duodenal perforation	2 (3.7)
Mast cell tumor prophylaxis	1 (1.9)
NSAID toxicosis	4 (7.4)

### 3.3 Outpatient prescription patterns

Of the dogs surviving to discharge, 54% (95/175) were discharged with owner instructions to continue to administer a PPI PO. Within the population of dogs with an identified appropriate indication for use, 59% (32/54) of dogs were prescribed a PPI PO, 22% (12/54) of dogs were not prescribed a PPI, and 19% (10/54) did not survive to discharge. Of the dogs with an adequate indication for use that were not prescribed a PPI, 17% (2/12) were prescribed a H_2_‐receptor antagonist (H2RA). Of the dogs that were discharged with owner instructions to continue to administer a PPI PO, 52% (49/95) were prescribed an inadequate dose, 92% (87/95) had no directions or inadequate directions for administration, 52% (50/95) received no written directions for discontinuation and 0% (0/95) received instructions to taper the medication before discontinuation. The prescribed dosages are summarized in Table 3.

**TABLE 3:** Dosage and frequency of administration categories and distribution among 95 dogs prescribed a PPI at discharge

Prescribed dosage of PPI at discharge	Prescriptions (n [%]) n = 95
<0.5 mg/kg q12h	0 (0)
<0.5 mg/kg q24h	1 (1.1)
0.5‐0.9 mg/kg q12h	19 (20.0)
0.5‐0.9 mg/kg q24h	21 (22.1)
≥1.0 mg/kg q12h	25 (26.3)
≥1.0 mg/kg q24h	27 (28.4)
No dose listed	2 (2.1)

### 3.4 Co‐prescribed medications

Of the dogs discharged with a PPI, 100% (95/95) of dogs were discharged with additional medications. The number of co‐prescribed drugs ranged from 1 to 10, with a median of 4 prescriptions. Of the dogs discharged with a PPI, 70% (67/95) were prescribed an anti‐nausea or an anti‐emetic medication or both, 13% (12/95) were prescribed a pro‐kinetic, 33% were prescribed an additional gastro‐protectant to include sucralfate (29/95), misoprostol (0), or antacids (5/95), 72% (68/95) were prescribed antibiotics, 7% (7/95) were prescribed an appetite stimulant, 24% (23/95) were prescribed a corticosteroid, 9.5% (9/95) were prescribed a secondary immunosuppressive medication, 11% (10/95) were prescribed an NSAID, 36% (34/95) were prescribed an alternative analgesic, 7% (7/95) were prescribed an anti‐platelet medication, 5% (5/95) were prescribed an anti‐coagulant medication, and 41% (39/95) were prescribed a medication that did not fall within the listed drug classes.

## 4 DISCUSSION

Our retrospective study of a single tertiary care facility describes the administration patterns of PPIs to hospitalized patients over a 5‐year time period. In humans, inappropriate use of PPIs has been well documented and includes inadequate indications for use, inappropriate duration of treatment, and unclear instructions for discontinuation of the medication on an outpatient basis. It is estimated that up to 65% of human patients prescribed PPIs have no documented ongoing indication for use,^3^ and it is possible that this number is underestimated given that PPIs are available over the counter. Furthermore, chronic PPI use has been associated with a number of adverse events including acute kidney injury,^10^, ^11^ osteoporotic fractures,^12^ hypocobalaminemia,^13^ hypomagnesemia,^14^ and dementia.^15^ Several studies describe the frequency and type of adverse effects associated with PPI administration in dogs.^5^, ^6^, ^8^ Despite these studies, inappropriate use of these medications is as likely or more common than observed in human medicine. For example, in a recent survey of small animal general practitioners in Portugal, 98.3% had prescribed a PPI without an appropriate indication for use.^16^

Gastric acid secretion is a normal physiologic mechanism that contributes to appropriate digestion of protein, release of cobalamin, absorption of inorganic iron^17^ and calcium,^18^ and regulation of the intestinal microbiome by suppression of bacterial overgrowth.^19^ The stomach has natural protective mechanisms to prevent against gastric erosion and ulceration as a result of gastric acid secretion and therefore acid suppression generally is not recommended in the absence of erosive or infiltrative disease.^20^, ^21^, ^22^, ^23^, ^24^ Appropriate indications for PPI administration to dogs include prophylaxis for gastric hyperacidity‐mediated GUE, treatment of esophagitis, and to promote healing of gastric and proximal duodenal lesions resulting in GI bleeding. Gastric hyperacidity in dogs primarily has been reported in cases of neoplasia (eg, mast cell disease,^25^ gastrinoma) but additional further studies are needed to fully characterize whether hyperacidity occurs with other disease processes. In our study, only 27% of dogs prescribed a PPI had an identifiable appropriate indication for use.

A higher proportion of dogs was prescribed a PPI with no appropriate indication for use, representing 68.5% of patients. The reasons for inappropriate use of PPIs in hospitalized dogs are likely multi‐factorial. Routine administration of PPIs seems to occur frequently in the hospital setting, including for the treatment of nausea, vomiting, or both despite no pharmacologic evidence for an anti‐nausea or anti‐emetic effect of PPIs.^7^ These conditions include nonerosive gastritis, pancreatitis, and hepatic and renal injury. Proton pump inhibitors also often are used as prophylaxis against GUE for diseases in which the benefit of PPI use (eg, non‐hyperacidity diseases) is largely unknown as well as in combination with NSAIDs, which is not currently recommended.^5^, ^8^ The use of these medications in absence of physical examination and clinicopathologic evidence of ongoing ulceration and GI bleeding is not recommended.^9^

The effective dose of omeprazole in dogs to achieve gastric pH goals known to result in healing of upper GI ulceration and erosion in humans has been suggested to be 1.0 mg/kg PO q12h, although recent evidence suggests that 0.5 mg/kg is effective as long as it is administered q12h.^26^, ^27^ In our study, 52% of dogs were prescribed an inadequate dose, raising concern for ineffective treatment. It is possible that this percentage at least partially reflects the timeframe from which the records were selected compared to the date with which the ACVIM consensus statement on the rational use of gastro‐protectants was published. Last, of the dogs that were discharged with client instructions to continue a PPI PO, 92% of dogs had no directions or inadequate directions for administration and no dogs had directions to taper the medication before discontinuation, which could represent lack of understanding of the pharmacologic properties, affect overall efficacy of treatment, and lead to adverse effects associated with gastric acid hypersecretion after abrupt discontinuation of treatment.

Of those surviving to discharge, 54% of dogs that were administered a PPI in hospital were not discharged with a PPI, which may suggest inappropriate use of this medication given the current standards of treatment. Given that time to onset of maximal efficacy of PPIs is 2 to 4 days^28^ and the recommended duration of treatment for upper GI ulceration and esophagitis in people ranges from 4 to 12 weeks,^29^, ^30^ this finding alone is suggestive of inappropriate use and potentially suggests that the medication was discontinued before even reaching maximal efficacy. If clinical suspicion was high enough to warrant the prescription of a PPI, then the majority of dogs also should have been discharged with instructions to continue a PO equivalent. Last, only 59% of dogs that had an identified reason for PPI prescription in hospital were discharged with PPI treatment, which further reflects an overall lack of rational use.

Inappropriate medication use also represents a misuse of financial resources. In the United States, expenditure on PPIs is estimated at over 11 billion dollars annually.^31^ At our institution, a standard vial of pantoprazole containing 40 mg with the dispensing fee costs $10.84, the equivalent of 24 hours of standard treatment for a 20 kg dog. Given that finances can have a substantial impact on the decision to continue veterinary care, the rationale and proposed benefit of each selected medication should be carefully considered.

Dogs hospitalized at tertiary care facilities commonly are discharged with recommendations to administer multiple medications, raising concern for medication interactions as well as poor compliance. Prescription of multiple medications potentially could be limited by rational selection. The average number of medications that dogs were discharged with in addition to a PPI in our study was 4.4, with some dogs being discharged with an additional 10 medications. In studies of human patients, decreases in medication compliance are associated with increasing complexity of treatment regimen, and it is estimated that 50% of patients do not take their medications as prescribed.^32^ Given the added challenge of medication administration to sick dogs, the number of medications prescribed is likely a contributing factor to inadequate medication compliance in veterinary medicine,^33^, ^34^, ^35^ which can directly impact patient outcome. Last, multiple medication interactions with PPIs have been identified in human medicine, although this situation still needs to be characterized in veterinary medicine.

The limitations of our study include its retrospective nature. Portions of the dog's history, physical examination findings, and progress in hospital may not have been adequately documented in the written medical record, precluding identification of an adequate indication for use. Furthermore, the percentage of prescriptions without discontinuation instructions could be overestimated given that the prescribing clinician could have intended for the client to discontinue omeprazole once the original number of tablets had been administered.

The evaluation of inpatient prescriptions of PPIs at a tertiary care facility identified that the majority were prescribed with an inappropriate indication for use. Furthermore, hospitalized dogs that received PPIs frequently were not provided a PPI on discharge, raising concern for lack of rational drug use given recommended duration of treatment for suspected gastric‐acid related disorders. The prescribing patterns in those dogs that were discharged with PPIs also indicated a high prevalence of inadequate doses to meet criteria for gastric acid suppression. Last, our evaluation identified a high number of concurrently prescribed medications, which may raise concern for an inverse relationship with client compliance with regard to medication administration. An additional study to evaluate whether the ACVIM consensus statement guidelines have since altered practices at our institution would be advisable.

## CONFLICT OF INTEREST DECLARATION

Authors declare no conflict of interest.

## OFF‐LABEL ANTIMICROBIAL DECLARATION

Authors declare no off‐label use of antimicrobials.

## INSTITUTIONAL ANIMAL CARE AND USE COMMITTEE (IACUC) OR OTHER APPROVAL DECLARATION

Authors declare no IACUC or other approval was needed.

## HUMAN ETHICS APPROVAL DECLARATION

Authors declare human ethics approval was not needed for this study.
